# Pulsed moxifloxacin for the prevention of exacerbations of chronic obstructive pulmonary disease: a randomized controlled trial

**DOI:** 10.1186/1465-9921-11-10

**Published:** 2010-01-28

**Authors:** Sanjay Sethi, Paul W Jones, Marlize Schmitt Theron, Marc Miravitlles, Ethan Rubinstein, Jadwiga A Wedzicha, Robert Wilson

**Affiliations:** 1 Division of Pulmonary, Critical Care and Sleep Medicine, University of Buffalo, State University of New York, Buffalo, NY, USA; 2 Division of Cardiac and Vascular Services, St George's, University of London, UK; 3 Clinical Operations, Bayer Pty Ltd, Isando, Johannesburg, South Africa; 4 Fundació Clínic, Institut d'Investigacions Biomèdiques August Pi i Sunyer (IDIBAPS), Barcelona, Spain; 5 Section of Infectious Diseases, University of Manitoba Faculty of Medicine, Winnipeg, MB, Canada; 6 Academic Unit of Respiratory Medicine, The Royal Free and University College Medical School, London, UK; 7 Respiratory Medicine, Royal Brompton Hospital, London, UK

## Abstract

**Background:**

Acute exacerbations contribute to the morbidity and mortality associated with chronic obstructive pulmonary disease (COPD). This proof-of-concept study evaluates whether intermittent pulsed moxifloxacin treatment could reduce the frequency of these exacerbations.

**Methods:**

Stable patients with COPD were randomized in a double-blind, placebo-controlled trial to receive moxifloxacin 400 mg PO once daily (N = 573) or placebo (N = 584) once a day for 5 days. Treatment was repeated every 8 weeks for a total of six courses. Patients were repeatedly assessed clinically and microbiologically during the 48-week treatment period, and for a further 24 weeks' follow-up.

**Results:**

At 48 weeks the odds ratio (OR) for suffering an exacerbation favoured moxifloxacin: per-protocol (PP) population (N = 738, OR 0.75, 95% confidence interval (CI) 0.565-0.994, p = 0.046), intent-to-treat (ITT) population (N = 1149, OR 0.81, 95% CI 0.645-1.008, p = 0.059), and a *post-hoc *analysis of per-protocol (PP) patients with purulent/mucopurulent sputum production at baseline (N = 323, OR 0.55, 95% CI 0.36-0.84, p = 0.006).

There were no significant differences between moxifloxacin and placebo in any pre-specified efficacy subgroup analyses or in hospitalization rates, mortality rates, lung function or changes in St George's Respiratory Questionnaire (SGRQ) total scores. There was, however, a significant difference in favour of moxifloxacin in the SGRQ symptom domain (ITT: -8.2 vs -3.8, p = 0.009; PP: -8.8 vs -4.4, p = 0.006). Moxifloxacin treatment was not associated with consistent changes in moxifloxacin susceptibility. There were more treatment-emergent, drug related adverse events with moxifloxacin vs placebo (p < 0.001) largely due to gastrointestinal events (4.7% vs 0.7%).

**Conclusions:**

Intermittent pulsed therapy with moxifloxacin reduced the odds of exacerbation by 20% in the ITT population, by 25% among the PP population and by 45% in PP patients with purulent/mucopurulent sputum at baseline. There were no unexpected adverse events and there was no evidence of resistance development.

**Trial registration:**

ClinicalTrials.gov number, NCT00473460 (ClincalTrials.gov).

## Background

The morbidity and mortality of chronic obstructive pulmonary disease (COPD) is substantially contributed to by frequent acute exacerbations of the disease. Higher exacerbation rates have been related to a faster decline in lung function [1-3], and a larger reduction in quality of life [4]. In addition, mortality has been shown to increase with frequent, severe exacerbations, particularly if these require hospitalization [5]. Exacerbations account for between one-third to one-half of the health economic burden of COPD [6,7]. Reducing exacerbations is now one of the goals of COPD maintenance treatment, and long-acting bronchodilators and inhaled corticosteroids have been moderately efficacious in this regard [8].

The role of respiratory bacterial pathogens in COPD has been clarified in recent years. It is likely that 40-50% of exacerbations are related to bacterial infection, particularly strains that are new to the patient [9]. This engenders airway and systemic inflammation that results in worsening airway physiology, ultimately manifesting as respiratory and systemic symptoms [10,11]. The presence of bacteria in the airways in stable COPD, long thought to be innocuous, may also contribute to COPD pathogenesis by causing inflammation and thereby structural airway damage [12-16].

It is possible, therefore, that reduction of airway bacterial load and/or prevention of new strain acquisition in COPD patients by the use of antibiotics may reduce the frequency and severity of exacerbations. Results of previous trials of prophylactic use of antibiotics in COPD conducted prior to 1970 demonstrate inconsistent and small benefits of such therapy. However, these studies were limited by the small numbers of patients included, the use of low doses of narrow-spectrum antibiotics, and by inadequate efficacy assessment [17]. Though widely used for treatment, respiratory fluoroquinolones have not been investigated in the prevention of COPD exacerbations. These drugs have several characteristics that make them attractive as prophylactic agents in COPD, such as potent *in-vitro *antimicrobial activity against the major pathogens in COPD, excellent penetration into respiratory tissues, high oral bioavailability, and proven efficacy in the treatment of exacerbations, including increasing the exacerbation-free interval [18,19].

Therefore, the PULSE study was conducted to determine whether intermittent pulsed therapy with the respiratory fluoroquinolone, moxifloxacin, was more efficacious than placebo in the reduction of exacerbations of COPD.

## Methods

### Patients

Stable COPD patients recruited for this study had to be at least 45 years of age, have smoked for at least 20 pack years, had a ratio of pre-bronchodilator forced expiratory volume in 1 second (FEV_1_) to forced vital capacity (FVC) ≤ 0.70 as well as a percent predicted forced expiration volume in 1 second (PFEV_1_) ≤ 80%. In addition, they had to have chronic bronchitis as defined by the American Thoracic Society [20] and at least two exacerbations requiring treatment with antibiotics and/or oral steroids in the 12 months prior to enrolment. Further inclusion and exclusion criteria are provided in Additional file 1: Inclusion and exclusion criteria. Pre-specified protocol violations resulted in patients being excluded from the per-protocol (PP) population (see Additional file 2: Pre-specified protocol violations). The study was approved by local ethics review committees and all patients gave written informed consent.

### Study design

PULSE was a randomized, double-blind, placebo-controlled, parallel group, multicentre international clinical trial that was conducted at 76 centres in 15 countries. Within 1 week of screening, eligible patients were randomly assigned to treatment with either moxifloxacin 400 mg orally once daily for 5 days or matching placebo for 5 days. Treatment was repeated every 8 weeks for a total of 6 courses. After randomization, patients were seen every 8 weeks for clinical evaluation to record any exacerbations and adverse events, and to dispense the medication course. Adherence to treatment was assessed by collecting and counting remaining pills at the subsequent study visit. At each visit, spirometry was performed and disease-related health status was assessed by using the St George's Respiratory Questionnaire (SGRQ). An end-of-treatment (EOT) visit was performed at 48 weeks after randomization. Three additional follow-up visits at 8-weekly intervals were conducted for a total study duration of 72 weeks.

Clinical laboratory assessments, including blood chemistry and haematology, were performed pre-treatment, 24 weeks after randomization, and at the end of treatment.

Patients were requested to continue with their usual medication during the study, including long-acting bronchodilators and inhaled steroids; any adjustment made to this therapy during the study was one reason for exclusion from the PP population. Patients were instructed to continue with study medication in the event of an exacerbation.

### Outcome measurements

The primary efficacy end point was the frequency of exacerbations during the treatment period, from randomization to the EOT visit at 48 weeks. The primary definition of exacerbation was an extended definition added before unblinding to maximize clinically relevant events. This definition included any confirmed acute exacerbation of chronic bronchitis (AECB) or unconfirmed pneumonia or any other lower respiratory tract infection (LRTI) with the exception of confirmed pneumonia, all requiring intervention (start of systemic antibiotic and/or start of systemic steroid and/or hospitalization within 7 days of the start date of exacerbation) and with a minimum of 2 weeks between the start of two consecutive exacerbations.

The secondary definition included any confirmed AECB (but excluded confirmed/unconfirmed pneumonia and any other LRTIs), minimally 2 weeks between start of two consecutive exacerbations whether or not intervention was documented.

Definitions of AECB and confirmed and unconfirmed pneumonia are given in Additional file 3: Definitions of exacerbations.

Secondary efficacy outcomes included hospitalization and mortality, changes in disease-related health status assessed with the SGRQ and changes in lung function measured as %PFEV_1_.

Adverse events (coded using the MedDRA system) and medications were collected at each study visit. Study staff and patients' regular physicians were instructed to treat exacerbations during the study period with non-fluoroquinolone antibiotics with or without a short course of oral steroids.

### Bacteriological assessments

Sputum samples were collected from all patients at all clinic visits and were processed locally for Gram stain and culture. For the following organisms, defined in the protocol as potential respiratory pathogens in COPD, moxifloxacin susceptibility testing was performed locally by E-test: *Haemophilus influenzae*, *Haemophilus parainfluenzae*, *Streptococcus pneumoniae, Moraxella catarrhalis, Klebsiella pneumoniae*, *Pseudomonas aeruginosa*, and *Staphylococcus aureus*.

Monitoring of changes in gastrointestinal flora was conducted by collecting rectal swabs from a subgroup of 211 patients in 23 sites at baseline, week 24, week 48 (EOT), and week 72. *Staphylococcus *spp., *Escherichia coli*, *Enterococcus *spp., *Enterobacter *spp., *K. pneumoniae*, *P. aeruginosa *and *Candida *spp. were isolated from the rectal swab cultures. All bacterial isolates were processed locally and then transported to a central laboratory where isolates were re-identified and the minimum inhibitory concentrations (MIC) to a range of antibiotics were determined by broth microdilution, according to Clinical Laboratory Standards Institute (CLSI) recommendations.

### Analysis populations

This trial was designed as a proof-of-concept study to test whether long-term intermittent treatment with an antibiotic would reduce exacerbation frequency in patients with COPD. To avoid potential dilution of the definitive efficacy results, the primary population for efficacy analyses was the PP population, as defined in Additional file 4: Statistical analysis. Analyses in the intent-to-treat (ITT) population, randomized patients who received at least one dose of study medication, were supportive.

Pre-specified patient subgroups for analysis included those with: use of inhaled steroids at any time during the study; use of systemic steroids at any time during the study; use of long-acting bronchodilators at any time during the study; past smokers at baseline; current smokers at baseline; patients with a baseline 50 < %PFEV_1 _≤ 80; patients with a baseline 30 < %PFEV_1 _≤ 50; patients with a baseline %PFEV_1 _≤ 30; patients with medication violations during the 48-72-week period of the study. *Post-hoc *subgroup analyses of patients with mucopurulent/purulent sputum and those without mucopurulent/purulent sputum at baseline were also carried out. The assessment of sputum purulence was made by the investigator based on patient report of colour of sputum.

### Statistical analysis

Continuous demographic variables were analysed using two-way analysis of variance (ANOVA) and categorical demographic variables by a Cochran-Mantel-Haenszel test. A p-value of < 0.05 was considered significant. Further information on statistical analysis is given in the Additional file 4: Statistical analysis.

The primary efficacy variable was the number of exacerbations recorded after 48 weeks of intermittent pulsed therapy. Numbers of exacerbations were grouped into four categories: 0, 1, 2, and ≥ 3, and a pre-specified logistic regression model was used to test the null hypothesis that the number of exacerbations in the moxifloxacin group was not different from the number of exacerbations in the placebo group. Odds ratios, adjusted for region and %PFEV_1_, were calculated for the mean rate of exacerbations. The absolute risk reduction was also tested, to calculate the number of patients needed to treat (NNT) to prevent one exacerbation.

Secondary efficacy outcomes were analysed as follows. Hospitalization and mortality were analysed by Fisher's exact test. Responses to the SRGQ were analysed using an analysis of covariance (ANCOVA) model, with the null hypothesis of no change in total SGRQ score between baseline and EOT, and adjusted for geographical region, SGRQ total score at baseline, sex, age, and treatment. Changes in lung function measured by %PFEV_1 _were analysed using a repeated measures ANOVA adjusted for region, visit, treatment group, and the interaction term between treatment and visit.

## Results

### Study population

**Figure 1:**
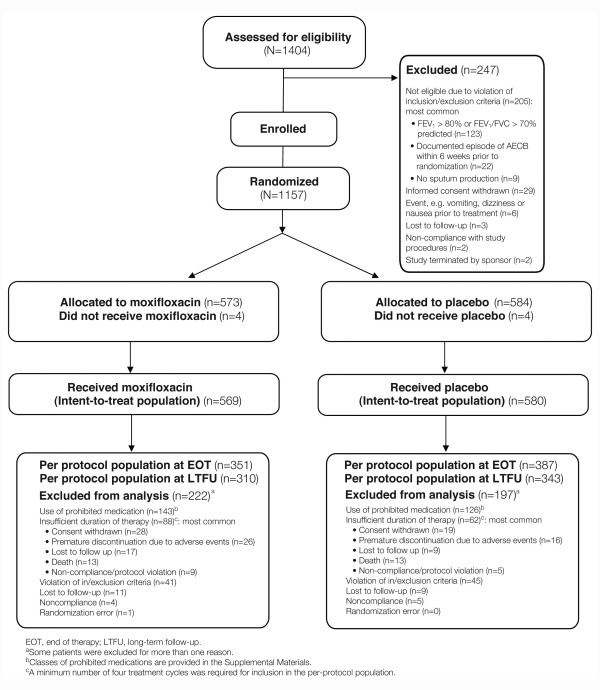
**Progression of patients through the study**.

A CONSORT flow diagram charts the disposition of patients throughout the study (Figure 1). A total of 1404 eligible patients were enrolled. Of these, 1157 were randomized to either moxifloxacin (N = 573) or placebo (N = 584). Four patients in each group did not receive the study drug, leaving 569 and 580 patients in the ITT population for moxifloxacin and placebo, respectively. The primary analysis population was PP EOT, for which 351 and 387 patients in the moxifloxacin- and placebo-treated groups were eligible. Reasons for exclusion from the PP EOT population were similar between the moxifloxacin and placebo treatment arms (Figure 1). Demographic and baseline clinical characteristics of the ITT and PP EOT populations are shown in Additional file 5: Demographic, clinical and medical characteristics at baseline. There were no statistically significant differences in baseline characteristics between the ITT and PP EOT populations, or between patients randomized to moxifloxacin or placebo. Premature terminations during the 48-week study period were more frequent in the moxifloxacin group (n = 102, 17.8%) than in the comparator group (n = 78, 13.4%) (p = 0.036). The main reason for premature discontinuation was consent withdrawal in both treatment arms (n = 33, 5.8% and n = 28, 4.8% in the moxifloxacin- and placebo-treated patients, respectively).

### Frequency of exacerbations

#### Overall population

**Figure 2:**
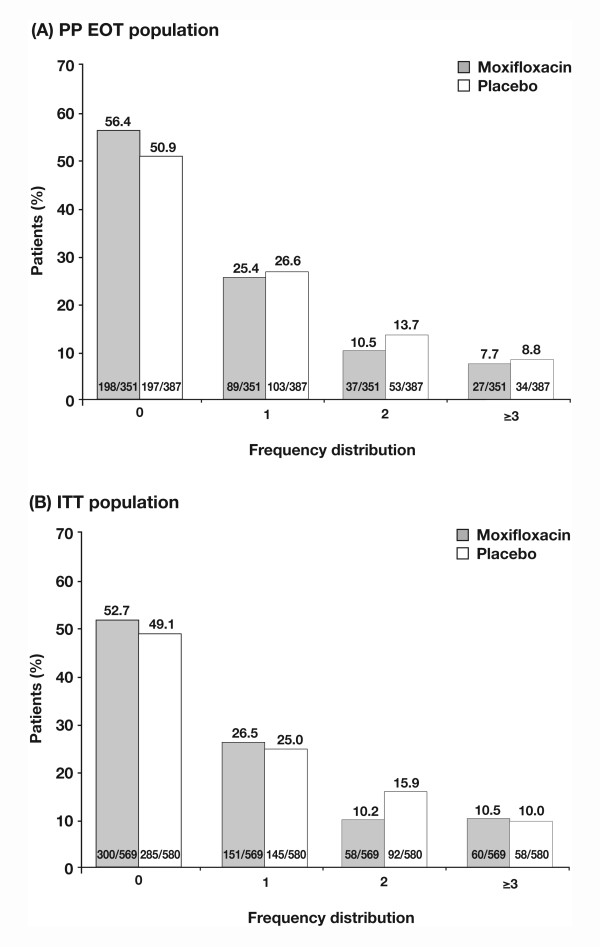
**Frequency distribution of exacerbations**. Data are after 48 weeks of intermittent therapy in (a) the per-protocol end-of-treatment (PP EOT) population and (b) the intent-to-treat (ITT) population.

**Figure 3:**
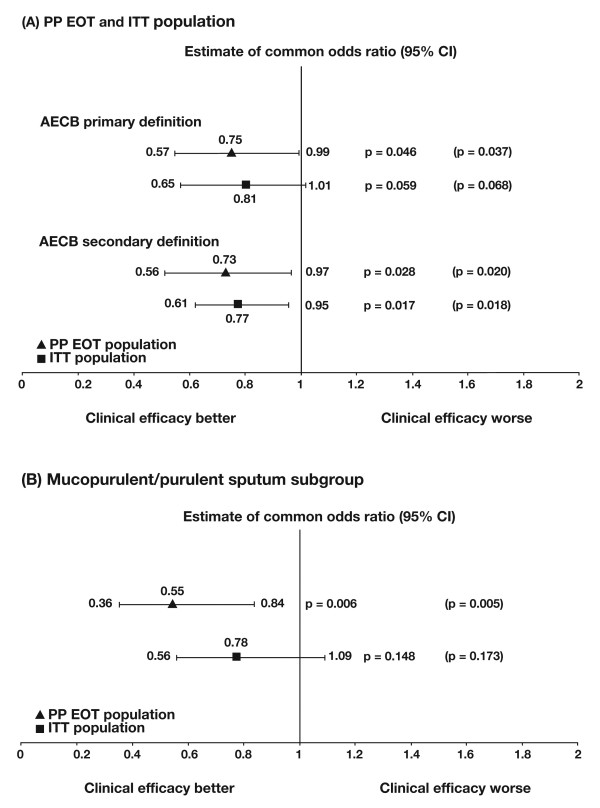
**Clinical efficacy of moxifloxacin vs placebo**. (a) Per-protocol end-of-treatment (PP EOT) and intent-to-treat (ITT) populations according to the primary and secondary definitions of an exacerbation, and (b) patients with purulent/mucopurulent sputum at baseline (PP EOT and ITT populations using the primary definition of an exacerbation). The first set of p-values on the graphs are from logistic regression analysis using the median value for patients missing at 48 weeks. Corresponding p-values for logistic regression analysis using last observation carried forward are given in brackets. AECB, acute exacerbation of chronic bronchitis.

Figures 2A and 2B illustrate the distribution of the number of exacerbations for the PP EOT and the ITT populations, respectively. In the PP EOT population, moxifloxacin reduced the likelihood of having an exacerbation by 25%. The mean rate of exacerbations was 0.88 (SD 1.24) in the placebo group and 0.75 (SD 1.15) in the moxifloxacin group, which resulted in a common odds ratio (OR) of 0.75 (95% confidence interval (CI) 0.57-0.99), and shows a statistically significant reduced odds for suffering an exacerbation with moxifloxacin treatment vs placebo (p = 0.046) (Figure 3a). At EOT there was an absolute risk reduction of 5.5% for exacerbations experienced during the 48-week study period. The NNT to prevent one exacerbation is 19. The reduction in exacerbations was similar when the secondary definition of exacerbation was applied. The mean (SD) rate of exacerbations was 0.96 (1.26) in the placebo group and 0.81 (1.16) in the moxifloxacin group, with an OR of 0.73 (95% CI 0.56-0.97; p = 0.028). The absolute risk reduction in the number of exacerbations experienced during the 48-week study period was 5.7% and the NNT to prevent one exacerbation was 18.

In the ITT population the mean (SD) rate of exacerbations (primary definition) was 0.94 (1.24) vs 0.88 (1.29) for placebo and moxifloxacin, respectively. Moxifloxacin reduced the likelihood of having an exacerbation by 19%, with an OR of 0.81 (95% CI 0.65-1.01; p = 0.059). The absolute risk reduction in exacerbations was 3.6 and the NNT was 28. A statistically significant difference in the reduction in the odds for experiencing an exacerbation with moxifloxacin was seen with the secondary definition (mean [SD] rate of exacerbations 1.04 [1.29] placebo, 0.94 [1.30] moxifloxacin; OR 0.77, 95% CI 0.61-0.95; p = 0.017) (Figure 3a). The absolute risk reduction for exacerbation was 4.9% and the NNT was 21. A trend for greater time to first exacerbation was seen for moxifloxacin- vs placebo-treated patients in both the PP EOT and ITT populations, although this did not reach statistical significance (p = 0.051 and 0.062 respectively) (see Additional file 6: Additional results, Figure S1).

### Subgroup analyses

#### Predefined efficacy subgroups

The ORs achieved for most of the predefined efficacy subgroups were similar to those for the overall PP and ITT populations (see Additional file 6: Additional results, Table S1). There were no significant differences between moxifloxacin and placebo in any of the efficacy subgroups in either the PP EOT or the ITT populations.

#### Patients with mucopurulent/purulent sputum

*Post-hoc *analysis of PP EOT patients (167 moxifloxacin, 156 placebo) who had mucopurulent/purulent sputum at baseline showed that in these patients moxifloxacin reduced the likelihood of exacerbations (primary definition) by 45% (mean [SD] rate of exacerbations: placebo 1.04 [1.29], moxifloxacin 0.79 [1.28]; OR 0.55; 95% CI 0.36-0.84; p = 0.006) (Figure 3b). For this subgroup, the absolute risk reduction in exacerbations was 14.4% and the NNT to prevent one exacerbation is 7. The reduction in odds of having an exacerbation was also significant when using the secondary definition: the mean (SD) rate of exacerbations was 1.11 (1.29) for placebo vs 0.84 (1.31) for moxifloxacin, resulting in an OR of 0.53 (95% CI 0.35-0.82; p = 0.004). This corresponds to a 15.2% absolute risk reduction in exacerbations and an NNT of 7. In the ITT population with mucopurulent/purulent sputum, the reduction in the odds of having an exacerbation with moxifloxacin was not statistically significant (Figure 3B).

#### Patients with non-mucopurulent/purulent sputum

*Post-hoc *analysis of PP EOT patients without mucopurulent/purulent sputum at baseline (n = 415, 184 moxifloxacin, 231 placebo) showed the mean (SD) rate of exacerbations using the primary definition was 0.77 (1.20) and 0.73 (1.02) for placebo and moxifloxacin, respectively, resulting in an OR of 0.96 (95% CI 0.66-1.40; p = 0.83). Similar results were seen using the secondary definition, where the mean (SD) rate of exacerbations was 0.86 (1.3) for placebo and 0.79 (1.02) for moxifloxacin resulting in an OR of 0.94 (95% CI 0.65-1.37; p = 0.76). In the ITT population without purulent/mucopurulent sputum, the reduction in the odds of having an exacerbation with moxifloxacin was also not statistically significant.

### Secondary efficacy results

#### Hospitalizations and mortality

The overall hospitalization rate in the PP EOT population was similar for moxifloxacin and placebo: 56/351 (15.9%) and 54/387 (14.0%), respectively (p = 0.80). In the ITT population there were more patients hospitalized in both groups (moxifloxacin: 131/569, 23.0%; placebo: 136/580, 23.4%), but this did not differ significantly between treatments. In addition, there were no statistically significant differences in frequency of hospitalization between the moxifloxacin- and placebo-treated groups of patients in either the PP EOT or the ITT populations, for the subgroups of COPD and LRTI-related hospitalizations, pneumonia-related hospitalizations or AECB-related hospitalizations (see Additional file 6: Additional results, Table S2).

The mortality rate during the 48 weeks of the study was low. In the PP EOT population there was 1/351 (0.3%) death in the moxifloxacin group and 3/387 (0.8%) deaths in the placebo group (see Additional file 6: Additional results, Table S2). Corresponding numbers for the ITT population were 15/569 (2.6%) in the moxifloxacin group and 17/580 (2.9%) in the placebo group. There were no significant differences in mortality rates between moxifloxacin and placebo in either population or any subgroup.

#### Lung function

Average lung function declined slightly during the study in both groups of patients. Lung function changes were similar for the moxifloxacin and placebo groups in both the ITT and PP EOT populations (see Additional file 6: Additional results, Tables S3 and S4).

#### Health status

Total scores on the SGRQ improved from baseline for both moxifloxacin and placebo. At Week 48 in the PP EOT population, the mean change from baseline total SGRQ score was -4.8 for moxifloxacin and -3.5 for placebo (p = 0.33) (see Additional file 6: Additional results, Table S5). Corresponding values in the ITT population were -4.0 for moxifloxacin and -2.8 for placebo (p = 0.29).

**Figure 4:**
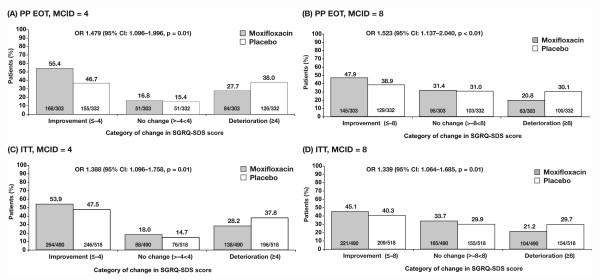
**Changes in St George's Respiratory Questionnaire symptom subscale scores**. (a) Per-protocol end-of-treatment (PP EOT) population using a minimum clinically important difference (MCID) of 4 units; (b) PP EOT population using an MCID of 8 units; (c) intent-to-treat (ITT) population using an MCID of 4 units; (D) ITT population using an MCID of 8 units.

The SGRQ domain scores of symptoms, activity and impact were also assessed (see Additional file 6: Additional results, Table S6). Categorical responder analysis of the SGRQ symptom domain scores showed a statistically significant difference in favour of moxifloxacin in the number of patients showing at least a 4-point improvement and at least an 8-point improvement in both the PP EOT and ITT populations (p ≤ 0.01) (Figure 4). Moxifloxacin treatment did not result in significant improvement in the activity or impact subscores.

### Baseline bacteriology

#### Sputum isolates

Relevant respiratory pathogens were isolated at randomization from about 24% of all patients. The most common potential COPD pathogens in both the moxifloxacin and placebo groups were *H. influenzae*, *H. parainfluenzae*, and *S. pneumoniae *isolated from 8.3%, 6.6%, and 4.3% of the PP EOT population, respectively. *S. aureus, P. aeruginosa*, *M. catarrhalis*, and *K. pneumoniae *were isolated less frequently (from 2.6%, 2.4%, 2.0%, and 2.0%, of the PP EOT population, respectively).

**Figure 5:**
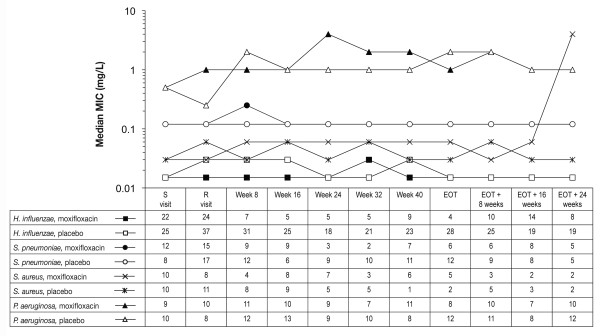
**Median MIC for moxifloxacin for *Haemophilus influenzae*, *Streptococcus pneumoniae*, *Staphylococcus aureus *and *Pseudomonas aeruginosa***. Data are shown for the entire 72 weeks of the study: 48 weeks of intermittent therapy and 24 weeks of follow-up. S, screening visit; R, randomization visit; EOT, end-of-treatment visit; MIC, minimum inhibitory concentration; numbers in the table are of patients with an isolate at a given time point.

Over the course of the 48-week treatment period, there was a trend towards a reduction in the total number of patients with pathogens isolated, which was more pronounced with moxifloxacin than placebo (data not shown, manuscript in preparation). Intermittent treatment with either moxifloxacin or placebo did not cause sustained MIC increases. The median MIC for moxifloxacin for *H. influenzae*, *H. parainfluenzae*, *M. catarrhalis*, *K. pneumoniae*, *S. pneumoniae *and *S. aureus *did not change or changed little during the study in both the placebo and moxifloxacin groups (Figure 5 and Additional file: Additional results, Table S7). In the moxifloxacin-treated group, one *S. pneumoniae *isolate resistant to moxifloxacin (MIC 4 mg/L) was isolated from a patient in the moxifloxacin arm on the sixth scheduled visit (Week 40); this strain was not associated with an exacerbation and did not persist at subsequent visits. For *S. aureus*, one to three moxifloxacin-resistant isolates were detected at baseline and at different points in the study; these isolates were not associated with exacerbations and did not persist. The median MIC of moxifloxacin against *P. aeruginosa *isolates increased to 4 mg/L at Week 24, but decreased to 1 mg/L by EOT, returning to value at randomization in the moxifloxacin group. In the placebo group, the median MIC of moxifloxacin against *P. aeruginosa *isolates increased from 0.5 mg/L at randomization to 2 mg/L by EOT.

#### Rectal isolates

For the monitored pathogens *Staphylococcus *spp., *E. coli*, *Enterococcus *spp., *Enterobacter *spp., *K. pneumoniae*, and *P. aeruginosa *isolated from the rectal swabs, no consistent major change in the median moxifloxacin MIC was detected in either treatment arm.

### Safety

#### Adverse events

The overall incidence of adverse events was similar in the moxifloxacin- (82.1%) and placebo-treated (85.0%) ITT/safety populations (Table 1). Treatment-emergent, drug-related adverse events were significantly higher with moxifloxacin as were premature discontinuations due to an adverse event, largely due to an excess of gastrointestinal disorders. The most common adverse events (occurring in ≥ 2 patients) leading to premature discontinuation of treatment, included nausea (moxifloxacin 5 vs placebo 0), vomiting (4 vs 0), diarrhoea (3 vs 1), hypersensitivity (2 vs 0), dyspnoea (2 vs 0), urticaria (2 vs 0) and upper abdominal pain (0 vs 2). One case of diarrhoea caused by *Clostridium difficile *was reported in the placebo group.

**Table 1:** Incidence of adverse events (intent-to-treat (ITT)/safety population).

	Moxifloxacin (N = 569) n (%)	Placebo (N = 580) n (%)	p-value*
**Any adverse event**	**467 (82.1)**	**493 (85.0)**	**0.181**

**Any treatment-emergent^a ^adverse event**	**258 (45.3)**	**265 (45.7)**	**0.906**

**Any treatment-emergent^a ^drug-related adverse events^b^**	**53 (9.3)**	**22 (3.8)**	**< 0.001**
Cardiac disorders	3 (0.5)	1 (0.2)	
Gastrointestinal disorders	27 (4.7)	4 (0.7)	
Diarrhea	17 (3.0)	9 (1.6)	
Nausea	6 (1.1)	0 (-)	
Vomiting	5 (0.9)	1 (0.2)	
General disorders and administration site conditions	4 (0.7)	2 (0.3)	
Asthenia	3 (0.5)	0 (-)	
Immune system disorders	4 (0.7)	0 (-)	
Hypersensitivity	3 (0.5)	0 (-)	
Infections and infestations	5 (0.9)	3 (0.5)	
Musculoskeletal and connective tissue disorders	3 (0.5)	1 (0.2)	
Nervous system disorders	6 (1.1)	4 (0.7)	
Dizziness	3 (0.5)	1 (0.2)	
Respiratory, thoracic and mediastinal disorders	8 (1.4)	0 (-)	
Dyspnea	4 (0.7)	0 (-)	
Skin and subcutaneous tissue disorders	5 (0.9)	5 (0.9)	
Deaths	15 (2.6)	17 (2.9)	

**Any treatment-emergent^a ^serious adverse event**	**94 (16.5)**	**97 (16.7)**	**0.926**

**Any treatment-emergent^a ^drug-related serious adverse event**	**9 (1.6)**	**3 (0.3)**	**0.076**

**Any adverse event leading to premature discontinuation**	**26 (4.6)**	**16 (2.8)**	**0.102**

**Any deaths**	**19 (3.3)**	**26 (4.5)**	**0.318**

^a^Events were determined to be treatment-emergent if they started after initiation of study medication up to 7 days post-therapy for each pulse of study medication

^b^Individual events listed under treatment-emergent drug-related adverse events occurring in ≥ 0.5% of subjects in either treatment group

*Post-hoc unadjusted Chi-squared test

## Discussion

Intermittent prophylactic pulsed treatment with moxifloxacin resulted in a 19% reduction in the odds of exacerbation in the ITT population and a 25% reduction in the PP EOT population in this study. This corresponds to NNTs of 28 and 19, respectively. Pre-specified subgroup analyses demonstrated that the reduction in exacerbations with moxifloxacin was seen in COPD of all severity categories, among smokers and ex-smokers, and in patients receiving concomitant COPD treatments including inhaled steroids and long-acting bronchodilators. In a *post-hoc *analysis, a larger (45%) reduction in the odds of exacerbation, corresponding to an NNT of 7, was seen among patients who reported purulent/mucopurulent sputum at baseline. The extent of reduction in exacerbations in these patients is similar to that seen with long-acting bronchodilators and inhaled steroids in recent clinical trials in COPD [21-26]. In a study with bronchoscopic sampling, sputum purulence was shown to be a reliable indicator of significant bronchial infection in patients with exacerbations of COPD [27]. It is possible that sputum purulence is also a marker for chronic bronchial infection in stable COPD, thereby explaining the greater benefit with prophylactic antibiotic treatment in these patients.

Intermittent moxifloxacin did not significantly improve overall health status, reduce rates of hospitalization or mortality, or slow the ongoing decline in lung function in COPD. The size and duration of the study were not adequate for these secondary end points. In the patient population recruited in this study, hospitalization and mortality rates were low, making it difficult to observe a difference between the two treatment groups. In terms of lung function, it is, perhaps, ambitious to expect improvements in underlying COPD in such a relatively short study of only six 5-day courses of an antibiotic.

There were more adverse events with moxifloxacin than with placebo, however, the overall levels of drug-related adverse events were low in both groups (9.3% with moxifloxacin and 3.8% with placebo) and comparable to findings in another large study with moxifloxacin in AECOPD [18]. The most common treatment-related adverse events related to the gastrointestinal tract, which also resulted in more premature terminations (3.6% vs 1.8%, in the moxifloxacin- and placebo-treated groups, respectively; p = 0.07). *C. difficile *infection was not reported in any of the patients taking moxifloxacin.

A major concern with antibiotic use is the emergence of resistant organisms. Regular monitoring of sputum flora and, in a subgroup, of faecal flora was therefore an essential part of the PULSE study. There was a transient increase in MIC of one strain of *S. pneumoniae *and three isolates of *S. aureus*. However, these isolates did not persist or cause exacerbations, and resistance emergence was not seen among patients in either moxifloxacin or placebo arms. The dosing regimen in PULSE was based on pharmacodynamic/pharmacokinetic principles, with intermittent dosing of a potent antibiotic at full doses, rather than use of therapeutic or subtherapeutic doses for prolonged periods. We speculate that such a dosing regimen was related to the lack of resistance emergence seen in this study although longer periods of observation are needed to confirm this. Patients who had colonization of moxifloxacin-resistant *P. aeruginosa *at baseline were excluded from this study but those with *P. aeruginosa *sensitive to moxifloxacin were included. The detection of *P. aeruginosa *with a higher MIC during the study than those present at screening/randomization suggests that patients colonized by this organism in the airway should not be considered for the therapeutic regimen described in this study. Further long-term studies are required to determine whether this type of intermittent preventive antibiotic therapy circumvents the development of resistant organisms.

There are three main limitations of the present study. The first is that there were fewer than expected exacerbations, despite the use of a wider definition of an exacerbation to maximize the number of events; only 0.88 were reported in the placebo group compared with the predicted 1.4 exacerbations per patient during the 48-week study period, with approximately 50% of the placebo group experiencing no exacerbations during 48 weeks. This 'placebo' effect of reduced exacerbations has been observed in other clinical trials and is probably related to closer monitoring and better compliance with maintenance COPD treatments [26]. Secondly, maintenance therapies for COPD, such as long-acting bronchodilators and inhaled steroids, which could affect frequency of exacerbations, were not standardized across all patients in this study. However, the frequency of such treatments did not differ between the treatment arms and benefit with moxifloxacin was seen in subgroups receiving such concomitant treatment. Finally, it is possible that some exacerbations were unreported due to the absence of daily monitoring [4,28].

The use of inhaled steroid and long-acting bronchodilators, both anticholinergic and beta-agonists, does result in a reduction in exacerbations. However, in a recent trial, when inhaled salmeterol/fluticasone was added to tiotropium, there was no additional reduction in frequency of exacerbations [29]. Current optimal therapy for COPD often does not result in an exacerbation-free patient. Increased incidence of pneumonia with inhaled steroids is an adverse effect when these drugs are used to reduce exacerbations [22,30,31]. Alternative approaches to reducing exacerbations are therefore required in some patients with COPD.

## Conclusions

Prevention of acute and chronic infection in COPD would be best achieved by augmenting the innate and adaptive immune responses with vaccines and novel drugs. Until this is possible, treatment with intermittent pulsed moxifloxacin could be indicated in certain subgroups of patients. Such patients include those with baseline purulent/mucopurulent or purulent sputum production, who are not colonized by *P. aeruginosa*, and who have an unacceptable frequency of exacerbations in spite of maximal therapy with inhaled agents for COPD or who experience complications such as pneumonia with these treatments. Though PULSE demonstrates that intermittent treatment with moxifloxacin is an effective option for preventing acute exacerbations in patients with COPD, further studies are required to determine the optimal patient population as well as dosing regimen and therapy duration for this approach.

## Competing interests

Sanjay Sethi has received research grants (26,000 USD) from Bayer HealthCare and honoraria for attendance at advisory boards and speaking work (23,000 USD) over the last 3 years.

Paul Jones has received honoraria (total 5,500 USD) from Bayer HealthCare AG for lecturing and attendance at advisory boards.

Marlize Schmitt Theron is a salaried employee of Bayer Pty Ltd.

Marc Miravitlles has received honoraria (total 11,000 USD) from Bayer HealthCare AG for consultancy and speaking work over the last two years.

Ethan Rubinstein has no conflicts of interest to disclose.

Jadwiga A. Wedzicha has received research grants (40,000 USD) from Bayer HealthCare and honoraria for attendance at advisory boards (10,000 USD).

Robert Wilson has received honorarium (total 18,900 Euro) from Bayer HealthCare AG for five advisory boards and four lectures over the last three years. Dr Wilson has also appeared as an expert witness for Bayer Healthcare AG.

## Authors' contributions

SS was the main author of the paper and undertook the initial drafting and assessment of data. All other authors contributed significantly to the design of the study, the collection and assessment of clinical data and development of this paper. MST was the Study Manager for Bayer Healthcare. All authors contributed significantly to the development of the manuscript, and all have all seen and approved the final version and take responsibility for the content.

## Supplementary Material

Additional file 1 **Inclusion and exclusion criteria**. List of criteria used to include or exclude patients from the trial. Click here for file

Additional file 2 **Pre-specified protocol violations**. Protocol violations that resulted in patients being excluded from the per-protocol population. Click here for file

Additional file 3 **Definitions of exacerbations**. Definitions of acute exacerbation of chronic bronchitis (AECB) and pneumonia. Click here for file

Additional file 4 **Statistical analysis**. Gives the model used for the statistical analysis and describes how missing data were handled. Click here for file

Additional file 5 **Demographic, clinical and medical characteristics at baseline**. Demographic data on the per-protocol and the intent-to-treat populations. Click here for file

Additional file 6 **Additional results**. Figure S1. Time to first exacerbation in (a) the PP EOT population and (b) the ITT population. Dropouts were treated as censored. PP EOT, per-protocol end-of-treatment; ITT, intent-to-treat. Table S1. Analysis of pre-specified subgroups. Data show number of exacerbations by the end of treatment in the per-protocol end-of-treatment (PP EOT) and intent-to-treat (ITT) populations. Table S2. Frequency of hospitalization and mortality. Data are shown for moxifloxacin- and placebo-treated patients, for the per-protocol end-of-treatment (PP EOT) and intent-to-treat (ITT) populations from Week 0 to Week 48. Table S3. Lung function. Data show the percentage predicted FEV_1 _during the 48 weeks of treatment with moxifloxacin or placebo in the per-protocol end-of-treatment (PP EOT) and intent-to-treat (ITT) populations. Table S4. Changes in lung function. Values are adjusted mean change of percentage predicted FEV_1 _over time in the per-protocol end-of-treatment (PP EOT) and intent-to-treat (ITT) populations. Table S5. Change from baseline in St George's Respiratory Questionnaire (SGRQ) total scores. Scores are shown for each visit for those patients in the per-protocol end-of-treatment (PP EOT) and intent-to-treat (ITT) populations who provided SGRQ data. Table S6. Changes in St George's Respiratory Questionnaire (SGRQ) symptom scores. Values show change from baseline to Week 48 in activity and impact subscores for the per-protocol end-of-treatment (PP EOT) and intent-to-treat (ITT) populations Table S7. Median moxifloxacin minimum inhibitory concentrations (MIC_50_). Values are MICs (numbers) for bacteria isolated from the sputum and rectal swab samples of moxifloxacin- or placebo-treated patients in the per-protocol end-of-treatment (PP EOT) population at each study visit. Click here for file
