# Correction: Pulsed moxifloxacin for the prevention of exacerbations of chronic obstructive pulmonary disease: a randomized controlled trial

**DOI:** 10.1186/1465-9921-11-88

**Published:** 2010-06-24

**Authors:** Sanjay Sethi, Paul W Jones, Marlize Schmitt Theron, Marc Miravitlles, Ethan Rubinstein, Jadwiga A Wedzicha, Robert Wilson

**Affiliations:** 1 Division of Pulmonary, Critical Care and Sleep Medicine, University of Buffalo, State University of New York, Buffalo, NY, USA; 2 Division of Cardiac and Vascular Services, St George's, University of London, UK; 3 Clinical Operations, Bayer Pty Ltd, Isando, Johannesburg, South Africa; 4 Fundació Clínic, Institut d'Investigacions Biomèdiques August Pi i Sunyer (IDIBAPS), Barcelona, Spain; 5 Section of Infectious Diseases, University of Manitoba Faculty of Medicine, Winnipeg, MB, Canada; 6 Academic Unit of Respiratory Medicine, The Royal Free and University College Medical School, London, UK; 7 Respiratory Medicine, Royal Brompton Hospital, London, UK

## 

Following the publication of this article [1], it was recognized that some important acknowledgements had been omitted. We would like to provide the corrected acknowledgements section below.
