# Supplementary material for: Pulsed moxifloxacin for the prevention of exacerbations of chronic obstructive pulmonary disease: a randomized controlled trial
Source: Respir Res. 2010 Jan 28;11(1):10. doi: 10.1186/1465-9921-11-10 (PMC2834642; doi:10.1186/1465-9921-11-10)
Supplement: Additional file 1 — Inclusion and exclusion criteria. List of criteria used to include or exclude patients from the trial. [file 1465-9921-11-10-S1.DOC]

### Additional file 1: Inclusion and exclusion criteria

### Inclusion criteria

- Male or female out-patients, aged ≥ 45 years
- Subjects suffering from chronic bronchitis (World Health Organization [WHO] Criteria—chronic bronchitis, defined as a cough productive of sputum on most days, for 3 consecutive months, for at least 2 consecutive years)
- FEV_1_ ≤ 80% and FEV_1_/FVC ≤ 70% predicted based on age, height, and sex
- No documented episode of acute exacerbation of chronic bronchitis (AECB) (requiring treatment) within 6 weeks of randomization and not experiencing an exacerbation at the time of screening
- Sputum production, on most days, even when exacerbation-free
- Presented with at least two documented (i.e. requiring antibiotics and/or systemic steroid administration) acute exacerbation episodes during the last 12 months
- If receiving chronic therapy with inhaled long-acting bronchodilators and/or inhaled or systemic steroids, the treatment must have remained stable for the proceeding 6 weeks prior to screening
- Smoking history of at least 20 pack-years
- Ability to complete questionnaires and diary as required
- Medical condition and social status compatible with study protocol procedures
- Subjects willing and able to give fully informed written consent

### Exclusion criteria

- Known hypersensitivity to moxifloxacin or other quinolones
- History of tendon disease/disorder (Amendment 1, 16 July 2004)
- Known congenital or documented-acquired QT prolongation; uncorrected hypokalemia; clinically relevant bradycardia; clinically relevant heart failure with reduced left ventricular ejection fraction; previous history of symptomatic arrhythmias; concomitant use of any of the following drugs, reported to increase the QT interval: antiarrhythmics class IA (e.g. quinidine, hydroquinidine, disopyramide) or antiarrhythmics class III (e.g. amiodarone, sotalol, dofetilide, ibutilide), neuroleptics (e.g. phenothiazines, pimozide, sertindole, haloperidol, sultopride), tricyclic antidepressive agents, certain antimicrobials (sparfloxacin, intravenous [IV] erythromycin, pentamidine, antimalarials particularly halofantrine), certain antihistaminics (terfenadine, astemizole, mizolastine), and others (cisapride, vincamine IV, bepridil, diphemanil)
- Female subjects who had been postmenopausal for less than 1 year, or not practicing an acceptable method of birth control (i.e. hormonal contraception, intra-uterine device, abstinence, or vasectomized partner), unless surgically incapable of childbearing. If a female subject of childbearing age was practicing an acceptable method of birth control (as mentioned above), she must have had a negative urine pregnancy test at randomization and at all subsequent visits during the treatment period prior to the dispensing of study medication (Amendment 1, 16 July 2004)
- Any known disease or condition with a life-expectancy of less than 24 months
- Severe hepatic impairment (Child–Pugh C) and/or a transaminase level > 5 times the upper limit of normal
- Receipt of an investigational drug within the last 30 days
- Known bronchial carcinoma, pulmonary tuberculosis, cystic fibrosis, documented chronic bronchial asthma, or diffuse bronchiectasis
- Active participation in intensive pulmonary rehabilitation programs
- Known history of chronic colonization of pathogenic organisms resistant to moxifloxacin (e.g. *Pseudomonas*, methicillin-resistant *Staphylococcus aureus*)
- Systemic or inhaled antibiotic therapy during the 6 weeks prior to screening and any long‑term antibiotic usage (Amendment 1, 16 July 2004)
- The need for home ventilatory support for COPD and subjects who had a tracheostomy *in situ* (subjects requiring home/portable oxygen therapy or continuous positive airway pressure (CPAP) for sleep apnea could be included)
- Unable to attend the specified visit dates as determined by individual visit calendars
