# Supplementary material for: Pulsed moxifloxacin for the prevention of exacerbations of chronic obstructive pulmonary disease: a randomized controlled trial
Source: Respir Res. 2010 Jan 28;11(1):10. doi: 10.1186/1465-9921-11-10 (PMC2834642; doi:10.1186/1465-9921-11-10)
Supplement: Additional file 2 — Pre-specified protocol violations. Protocol violations that resulted in patients being excluded from the per-protocol population. [file 1465-9921-11-10-S2.DOC]

### Additional file 2: Pre-specified protocol violations

- Violation of inclusion/exclusion criteria (primarily baseline lung function tests being out of range, insufficient smoking history, pretherapy exacerbation, co-morbidities that could affect outcome, age violation and requirement for ventilator support)
- Insufficient duration of therapy
- Non-compliance with study drug
- Consent withdrawal
- Lost to follow-up
- Use of prohibited concomitant medications (see below)

### Prior and concomitant therapy

Use of any investigational drug other than the study drug was not permitted. During the course of the study (treatment and follow-up periods), the administration of other systemic or inhaled antibacterial agents, for indications other than AECB, was not to exceed a total of 14 cumulative days. These treatments were to be reviewed on an individual basis and could have invalidated a subject at the end of the study. Fluoroquinolone antibiotics were not to be administered during the study; in such cases, the subject was to be deemed a protocol deviator. Despite such a deviation, however, the subject could continue in the study (Amendment 1, 16 July 2004).

Moxifloxacin was not to be administered concomitantly with antacids or other preparations containing magnesium or aluminum. Sucralfate and agents containing iron or zinc were to be administered at least 4 hours before or 2 hours after administration of an oral moxifloxacin dose.

Drugs that were to be avoided during the course of the study include those reported to increase the QT interval (see Exclusion criteria, above).

International Normalized Ratio monitoring was to be performed when anticoagulant therapy was taken concurrently with study drug and the oral anticoagulant dosage was to be adjusted, if necessary.

In addition, long-term inhaled long‑acting bronchodilators and/or inhaled/systemic steroids at screening were to be maintained on the same dose and regimen for the 6 weeks prior to study entry and until the end of the study. Any change was to be recorded as an adverse event of worsening of condition and the subject was to be regarded as a protocol deviator; despite these changes, however, the subject was to continue in the study. A narrative was to be written by the Investigator to explain the reasons for the change to baseline treatment (Amendment 1, 16 July 2004).

All concomitant medications were recorded in the Case Report Form, including trade name, daily dose (or range of doses), start and stop dates, and reason for administration.
