# Supplementary material for: Pulsed moxifloxacin for the prevention of exacerbations of chronic obstructive pulmonary disease: a randomized controlled trial
Source: Respir Res. 2010 Jan 28;11(1):10. doi: 10.1186/1465-9921-11-10 (PMC2834642; doi:10.1186/1465-9921-11-10)
Supplement: Additional file 3 — Definitions of exacerbations. Definitions of acute exacerbation of chronic bronchitis (AECB) and pneumonia. [file 1465-9921-11-10-S3.DOC]

### Additional file 3: Definitions of exacerbations

**AECB**

The definition of an acute exacerbation of chronic bronchitis (AECB) was the occurrence of each of the following three events:

1. Contact with a health care professional due to worsening of respiratory symptoms
2. (a) An increase in the intensity of at least two of the three signs/symptoms associated with exacerbations (sputum volume, dyspnoea, sputum purulence) as per Anthonisen criteria, or
   (b) An adverse event which indicates an AECB has occurred, i.e. an adverse event of AECB or related terms, pneumonia (with no infiltrate revealed on a chest X-ray) or respiratory failure which can be shown to be due to an AECB, or
   (c) Hospitalization due to an AECB or related term
3. Treatment with a systemic antibiotic and/or oral steroid.

**Definition of a confirmed and unconfirmed pneumonia**

Confirmed pneumonia: a diagnosis made by the investigator on the basis of signs and symptoms, which had to be confirmed by radiological findings consistent with pneumonia. Unconfirmed pneumonia: a diagnosis made by the investigator on the basis of signs and symptoms, but in the absence of a chest X-ray, or at least of a description of X-ray findings consistent with pneumonia
