# Supplementary material for: Pulsed moxifloxacin for the prevention of exacerbations of chronic obstructive pulmonary disease: a randomized controlled trial
Source: Respir Res. 2010 Jan 28;11(1):10. doi: 10.1186/1465-9921-11-10 (PMC2834642; doi:10.1186/1465-9921-11-10)
Supplement: Additional file 4 — Statistical analysis. Gives the model used for the statistical analysis and describes how missing data were handled. [file 1465-9921-11-10-S4.DOC]

### Additional file 4: Statistical analysis

**Statistical model**

**f(# of exacerbations) = α + β_1_ * treatment + β_2_ * region + β_3_ * baseline %PFEV_1_**

where f(# of exacerbations) was the logit link [ln(p/1–p)] used by SAS PROC LOGISTIC for logistic regression of ordinal variables.

**Handling missing data**

It was expected that some patients would drop out of the study before the 48-week time point. Every effort was made to follow such patients off-treatment to obtain the primary endpoint. Patients who dropped out were assigned to the median category from the patient’s treatment group, in the PP (EOT) population, for the primary AECB definition. The only exceptions to this were patients who already had more exacerbations than the median category at the time of dropout; for these patients, the observed number of exacerbations would be used.

A second analysis of missing data was also carried out in response to independent statistical review to ensure that the use of the median for missing data did not introduce bias. This was a last observation carried forward scheme where each dropout was placed in an exacerbation category depending on the last observation (0, >0 to ≤1, >1 to ≤2, >2). Here, a patient withdrawing at week 36 having experienced 1 exacerbation would be given the value 1 * 48/36 as an outcome.
