# Supplementary material for: Pulsed moxifloxacin for the prevention of exacerbations of chronic obstructive pulmonary disease: a randomized controlled trial
Source: Respir Res. 2010 Jan 28;11(1):10. doi: 10.1186/1465-9921-11-10 (PMC2834642; doi:10.1186/1465-9921-11-10)
Supplement: Additional file 5 — Demographic, clinical and medical characteristics at baseline. Demographic data on the per-protocol and the intent-to-treat populations. [file 1465-9921-11-10-S5.DOC]

### Additional file 5: Demographic, clinical and medical characteristics at baseline.

|  | PP EOT | | |  | ITT | | |
| --- | --- | --- | --- | --- | --- | --- | --- |
|  | Moxifloxacin  N = 351 | Placebo  N = 387 | p-value^*^ |  | Moxifloxacin  N = 569 | Placebo  N = 580 | p-value^*^ |
| Age, mean (SD) | 65.8 (8.9) | 66.2 (8.7) | 0.53 |  | 66.1 (8.9) | 66.6 (8.9) | 0.35 |
| Male sex, n (%) | 263 (74.9) | 291 (75.2) | 0.98 |  | 422 (74.2) | 428 (73.8) | 0.88 |
| Race white, n (%) | 257 (73.2) | 283 (73.1) | 0.57 |  | 426 (74.9) | 421 (72.6) | 0.57 |
| Smoking status |  |  | 0.32 |  |  |  | 0.35 |
| Current smokers, n (%) | 122 (34.8) | 121 (31.3) |  |  | 198 (34.8) | 187 (32.2) |  |
| Previous smokers, n (%) | 229 (65.2) | 266 (68.7) |  |  | 371 (65.2) | 393 (67.8) |  |
| Pack years of smoking | 53.6 (27.5) | 54.2 (28.8) | 0.84 |  | 54.0 (30.3) | 52.5 (29.9) | 0.38 |
| Concomitant medications, n (%) | 351 (100.0) | 383 (99.0) | 0.13 |  | 564 (99.9) | 573 (98.8) | 0.58 |
| Drugs for obstructive airways disease | 335 (95.4) | 369 (95.3) | 0.95 |  | 538 (94.6) | 551 (95.0) | 0.73 |
| Short acting bronchodilators | 242 (68.9) | 275 (71.1) | 0.53 |  | 406 (71.4) | 419 (72.2) | 0.74 |
| Long acting bronchodilators | 152 (43.3) | 167 (43.2) | 0.97 |  | 254 (44.6) | 264 (45.5) | 0.77 |
| Inhaled steroids | 136 (38.7) | 160 (41.3) | 0.47 |  | 235 (41.3) | 250 (43.1) | 0.54 |
| Theophylline | 102 (29.1) | 97 (25.1) | 0.22 |  | 165 (29.0) | 155 (26.7) | 0.39 |
| Systemic steroids | 1 (0.3) | 1 (0.3) | 1.00 |  | 2 (0.4) | 1 (0.2) | 0.62 |
| Others | 13 (3.7) | 22 (5.7) | 0.21 |  | 27 (4.7) | 33 (5.7) | 0.47 |
| Inhaled steroids / long-acting  bronchodilators | 91 (25.9) | 101 (26.1) | 0.96 |  | 144 (25.3) | 154 (26.6) | 0.63 |
| FEV_1_,  L, mean (SD)  % predicted, mean (SD) | 1.2 (0.5)  39.8 (13.9) | 1.2 (0.5)  41.3 (14.3) | 0.18  0.14 |  | 1.2 (0.5)  40.6 (15.6) | 1.2 (0.5)  42.2 (16.4) | 0.27  0.08 |
| FVC  L, mean (SD)  % predicted, mean (SD) | 2.7 (0.9)  70.9 (19.6) | 2.7 (0.8)  70.7 (16.6) | 0.78  0.91 |  | 2.7 (0.9)  70.6 (18.3) | 2.7 (0.8)  71.1 (18.1) | 0.98  0.68 |
| FEV_1_/FVC (%) | 44.3 (12.1) | 45.9 (12.0) | 0.07 |  | 45. (12.9) | 46.3 (12.3) | 0.10 |
| GOLD categories^†^  I  II  III  IV | 1 (0.3)  82 (23.4)  161 (45.9)  100 (28.5) | 3 (0.8)  94 (24.3)  192 (49.6)  96 (24.8) | 0.50 |  | 10 (1.8)  121 (21.3)  254 (44.6)  161 (28.3) | 10 (1.7)  131 (22.6)  277 (47.8)  146 (25.2) | 0.62 |
| Time (years) between diagnosis and entry, mean (SD) | 9.1 (6.9) | 9.1 (7.9) | 0.99 |  | 9.2 (7.1) | 9.2 (7.8) | 0.93 |
| Number of previous exacerbations in previous year, mean (SD) | 2.6 (1.0) | 2.6 (1.0) | 0.92 |  | 2.6 (1.0) | 2.7 (1.0) | 0.63 |
| Time (weeks) between last exacerbation and entry, mean (SD) | 18.4 (9.6) | 17.7 (8.8) | 0.36 |  | 17.9 (9.5) | 17.9 (9.0) | 0.96 |

^*^p-values for continuous variables are from ANOVA with treatment and region included as factors; p-values for categorical variables are from the Cochran–Mantel–Haenszel test with region included as a stratum.

^†^A small number of patients with FEV_1_ > 70% but < 80% were included in the study: PP EOT population, 7 (2.0%) and 2 (0.5%) in the moxifloxacin and placebo groups respectively; ITT population, 23 (4.0%) and 16 (2.8%) in the moxifloxacin and placebo groups respectively.

FEV_1_, forced expiratory volume in 1 second; FVC, forced vital capacity; GOLD, Global Initiative on Obstructive Lung Disease.
