# Supplementary material for: Pulsed moxifloxacin for the prevention of exacerbations of chronic obstructive pulmonary disease: a randomized controlled trial
Source: Respir Res. 2010 Jan 28;11(1):10. doi: 10.1186/1465-9921-11-10 (PMC2834642; doi:10.1186/1465-9921-11-10)
Supplement: Additional file 6 — Additional results. Figure S1. Time to first exacerbation in (a) the PP EOT population and (b) the ITT population. Dropouts were treated as censored. PP EOT, per-protocol end-of-treatment; ITT, intent-to-treat. Table S1. Analysis of pre-specified subgroups. Data show number of exacerbations by the end of treatment in the per-protocol end-of-treatment (PP EOT) and intent-to-treat (ITT) populations. Table S2. Frequency of hospitalization and mortality. Data are shown for moxifloxacin- and placebo-treated patients, for the per-protocol end-of-treatment (PP EOT) and intent-to-treat (ITT) populations from Week 0 to Week 48. Table S3. Lung function. Data show the percentage predicted FEV1 during the 48 weeks of treatment with moxifloxacin or placebo in the per-protocol end-of-treatment (PP EOT) and intent-to-treat (ITT) populations. Table S4. Changes in lung function. Values are adjusted mean change of percentage predicted FEV1 over time in the per-protocol end-of-treatment (PP EOT) and intent-to-treat (ITT) populations. Table S5. Change from baseline in St George's Respiratory Questionnaire (SGRQ) total scores. Scores are shown for each visit for those patients in the per-protocol end-of-treatment (PP EOT) and intent-to-treat (ITT) populations who provided SGRQ data. Table S6. Changes in St George's Respiratory Questionnaire (SGRQ) symptom scores. Values show change from baseline to Week 48 in activity and impact subscores for the per-protocol end-of-treatment (PP EOT) and intent-to-treat (ITT) populations Table S7. Median moxifloxacin minimum inhibitory concentrations (MIC50). Values are MICs (numbers) for bacteria isolated from the sputum and rectal swab samples of moxifloxacin- or placebo-treated patients in the per-protocol end-of-treatment (PP EOT) population at each study visit. [file 1465-9921-11-10-S6.DOC]

### Additional file 6: Additional results

**Figure S1:** Time to first exacerbation in (a) the PP EOT population and (b) the ITT population. Dropouts were treated as censored. PP EOT, per-protocol end-of-treatment; ITT, intent-to-treat.

**
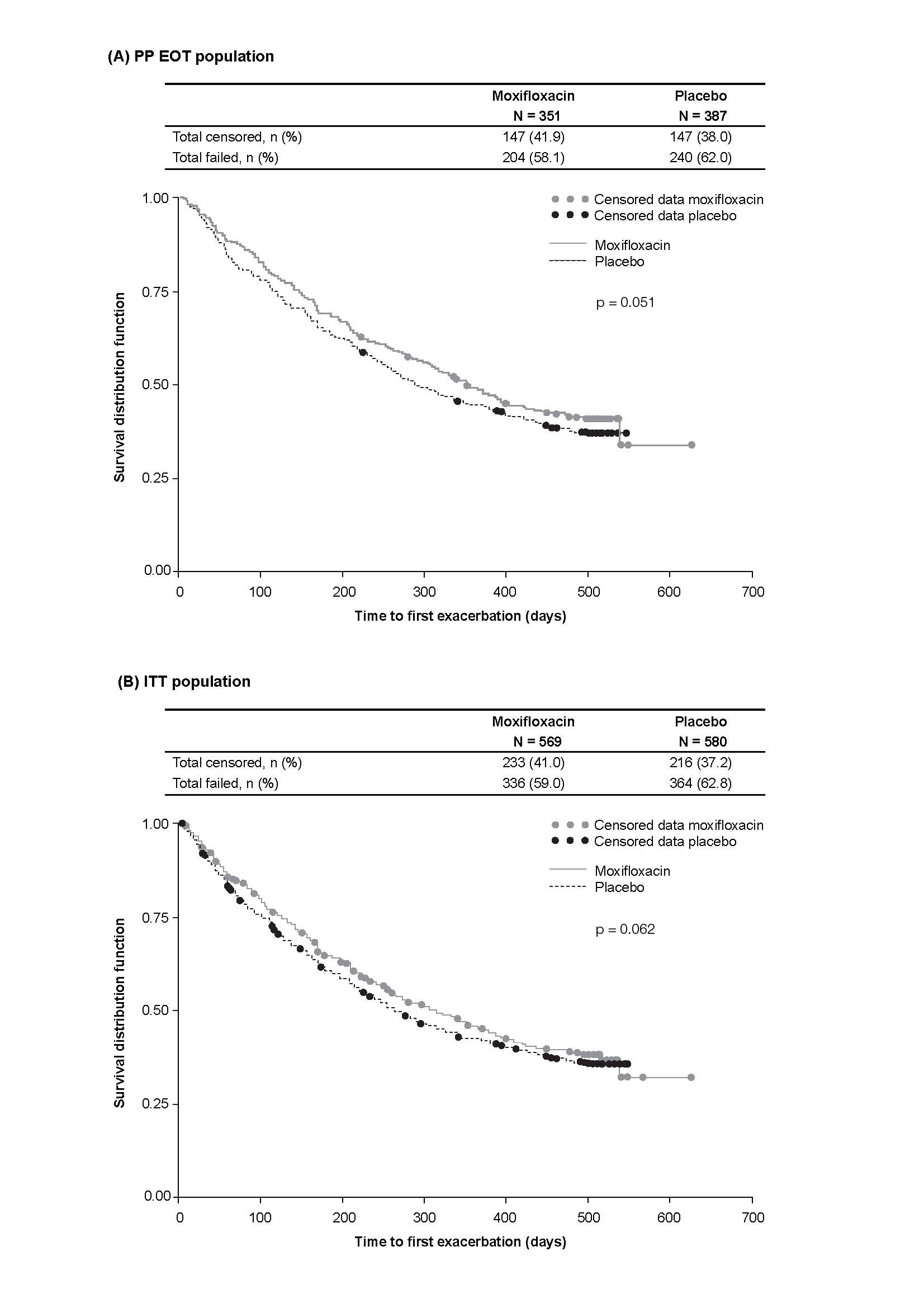
**

**Table S1.** Analysis of pre-specified subgroups. Data show number of exacerbations by the end of treatment in the per-protocol end-of-treatment (PP EOT) and intent-to-treat (ITT) populations.

| Patient subgroup | PP EOT | | | | |  | ITT | | | | |
| --- | --- | --- | --- | --- | --- | --- | --- | --- | --- | --- | --- |
|  | N_moxifloxacin_/  N_placebo_ | Odds ratio | 95% CI | p-value^a^ | p-value^b^ |  | N_moxifloxacin_/  N_placebo_ | Odds ratio | 95% CI | p-value^a^ | p-value^b^ |
| Inhaled steroids | 118/145 | 0.819 | 0.514, 1.305 | 0.401 | 0.384 |  | 214/247 | 0.842 | 0.595, 1.194 | 0.333 | 0.465 |
| Systemic steroids | 170/185 | 0.820 | 0.556, 1.208 | 0.316 | 0.224 |  | 296/285 | 0.803 | 0.596, 1.082 | 0.148 | 0.262 |
| Long-acting bronchodilator | 82/95 | 0.878 | 0.484, 1.592 | 0.668 | 0.633 |  | 147/161 | 0.842 | 0.546, 1.299 | 0.437 | 0.335 |
| 50 < %PFEV_1_ ≤ 80 | 82/94 | 0.775 | 0.416, 1.441 | 0.419 | 0.419 |  | 129/137 | 0.647 | 0.393, 1.065 | 0.087 | 0.091 |
| 30 < %PFEV_1_ ≤ 50 | 169/194 | 0.739 | 0.491, 1.111 | 0.146 | 0.140 |  | 272/279 | 0.806 | 0.585, 1.110 | 0.187 | 0.192 |
| %PFEV_1_ ≤ 30 | 100/97 | 0.685 | 0.396, 1.188 | 0.178 | 0.148 |  | 155/145 | 0.833 | 0.542, 1.279 | 0.403 | 0.459 |
| Medication violations during the 48–72-week period of the study | 41/44 | 1.227 | 0.552, 2.725 | 0.615 | 0.615 |  | 179/158 | 1.055 | 0.711, 1.565 | 0.790 | 0.517 |
| Past smokers at baseline | 229/266 | 0.752 | 0.529, 1.070 | 0.113 | 0.096 |  | 371/393 | 0.852 | 0.645, 1.124 | 0.256 | 0.327 |
| Current smokers at baseline | 122/121 | 0.744 | 0.453, 1.222 | 0.242 | 0.230 |  | 198/187 | 0.721 | 0.489, 1.064 | 0.099 | 0.085 |

PFEV_1_, predicted forced expiratory volume in 1 second.

^a^Logistic regression analysis using median value for patients missing at 48 weeks.

^b^Logistic regression analysis using last number carried forward for patients missing at 48 weeks.

**Table S2.** Frequency of hospitalization and mortality. Data are shown for moxifloxacin- and placebo-treated patients, for the per-protocol end-of-treatment (PP EOT) and intent-to-treat (ITT) populations from Week 0 to Week 48.

|  | PP EOT | | | ITT | | | |
| --- | --- | --- | --- | --- | --- | --- | --- |
|  | Moxifloxacin  (N = 351)  n (%) | Placebo  (N = 387)  n (%) | p-value^a^ |  | Moxifloxacin  (N = 569)  n (%) | Placebo  (N = 580)  n (%) | p-value^a^ |
| Frequency of hospitalization |  |  |  |  |  |  |  |
| Overall | 56 (15.95) | 54 (13.95) | 0.80 |  | 131 (23.02) | 136 (23.45) | 0.46 |
| COPD- and LRTI-related | 35 (9.97) | 34 (8.79) | 0.75 |  | 88 (15.47) | 93 (16.03) | 0.42 |
| Pneumonia-related | 5 (1.42) | 4 (1.03) | 0.79 |  | 17 (2.99) | 20 (3.45) | 0.39 |
| AECB-related | 27 (7.69) | 24 (6.20) | 0.82 |  | 60 (10.54) | 53 (9.14) | 0.81 |
| Mortality |  |  |  |  |  |  |  |
| Overall | 1 (0.28) | 3 (0.78) | 0.34 |  | 15 (2.64) | 17 (2.93) | 0.45 |
| COPD- and LRTI-related | 1 (0.28) | 2 (0.52) | 0.53 |  | 12 (2.11) | 12 (2.07) | 0.60 |
| Pneumonia-related | 0 | 0 | – |  | 4 (0.7) | 1 (0.17) | 0.97 |
| AECB-related | 0 | 0 | – |  | 0 | 1 (0.17) | 0.50 |

^a^Fisher’s exact test.

AECB, acute exacerbation of chronic bronchitis; COPD, chronic obstructive pulmonary disease; LRTI, lower respiratory tract infection.

**Table S3.** Lung function. Data show the percentage predicted FEV_1_ during the 48 weeks of treatment with moxifloxacin or placebo in the per-protocol end-of-treatment (PP EOT) and intent-to-treat (ITT) populations.

|  | PP EOT^a^ | |  | ITT^a^ | |
| --- | --- | --- | --- | --- | --- |
|  | Moxifloxacin  (N = 351)  Mean^b^ (SE) | Placebo  (N = 387)  Mean^b^ (SE) |  | Moxifloxacin  (N = 569)  Mean^b^ (SE) | Placebo  (N = 580)  Mean^b^ (SE) |
| Week 8 | 40.24 (0.796) | 41.42 (0.758) |  | 41.14 (0.715) | 42.17 (0.701) |
| Week 16 | 40.29 (0.788) | 41.98 (0.752) |  | 40.95 (0.713) | 42.79 (0.700) |
| Week 24 | 40.03 (0.791) | 41.00 (0.753) |  | 40.85 (0.729) | 42.31 (0.715) |
| Week 32 | 40.11 (0.759) | 40.79 (0.724) |  | 40.63 (0.695) | 41.87 (0.682) |
| Week 40 | 40.00 (0.777) | 40.70 (0.739) |  | 40.74 (0.710) | 41.66 (0.695) |
| Week 48 (EOT) | 39.01 (0.763) | 40.11 (0.727) |  | 39.79 (0.704) | 41.14 (0.690) |

^a^The p-values for these analyses come from the repeated measures ANOVA analyses. The p-value for treatment group was 0.2983 and 0.1660 for the PP (EOT) and ITT analyses respectively, i.e. no statistically significant difference between the treatment groups.

^b^The mean values are an estimate taken from the ANOVA model, adjusted for region, visit, treatment group and the interaction between treatment and visit.

SE, standard error.

**Table S4.** Changes in lung function. Values are adjusted mean change of percentage predicted FEV_1_ over time in the per-protocol end-of-treatment (PP EOT) and intent-to-treat (ITT) populations.

|  | PP EOT | | | |  | ITT | | | |
| --- | --- | --- | --- | --- | --- | --- | --- | --- | --- |
| Visit | N | Moxifloxacin | N | Placebo |  | N | Moxifloxacin | N | Placebo |
| Week 8 | 338 | 0.416 | 377 | 0.283 |  | 517 | 0.715 | 542 | 0.121 |
| Week 16 | 343 | 0.421 | 375 | 1.032 |  | 518 | 0.456 | 533 | 0.836 |
| Week 24 | 340 | 0.153 | 381 | –0.132 |  | 505 | 0.308 | 528 | 0.208 |
| Week 32 | 341 | 0.285 | 375 | –0.269 |  | 486 | 0.247 | 509 | –0.130 |
| Week 40 | 325 | 0.157 | 370 | –0.458 |  | 464 | 0.244 | 498 | –0.526 |
| Week 48 (EOT) | 325 | –0.839 | 359 | –0.994 |  | 462 | –0.694 | 487 | –0.977 |
| Week 56 | 316 | –0.831 | 359 | –0.465 |  | 452 | –0.414 | 482 | –0.378 |
| Week 64 | 314 | –1.094 | 345 | –0.597 |  | 449 | –0.778 | 466 | –0.476 |
| Week 72 | 303 | –1.110 | 335 | –0.974 |  | 433 | –0.642 | 457 | –0.738 |

**Table S5.** Change from baseline in St George’s Respiratory Questionnaire (SGRQ) total scores. Scores are shown for each visit for those patients in the per-protocol end-of-treatment (PP EOT) and intent-to-treat (ITT) populations who provided SGRQ data.

|  | PP EOT | | | | | |  | ITT | | | | | |
| --- | --- | --- | --- | --- | --- | --- | --- | --- | --- | --- | --- | --- | --- |
|  | Moxifloxacin | | | Placebo | | |  | Moxifloxacin | | | Placebo | | |
|  | N | Mean change | SD | N | Mean change | SD |  | N | Mean change | SD | N | Mean change | SD |
| Week 8 | 351 | –3.0 | 11.6 | 384 | –3.0 | 10.9 |  | 551 | –2.4 | 11.9 | 567 | –2.2 | 12.5 |
| Week 16 | 351 | –3.3 | 12.2 | 386 | –3.1 | 11.9 |  | 541 | –3.0 | 12.3 | 557 | –2.7 | 12.9 |
| Week 24 | 350 | –4.6 | 13.5 | 387 | –3.3 | 13.9 |  | 533 | –3.8 | 13.9 | 555 | –2.6 | 14.4 |
| Week 32 | 347 | –5.2 | 13.9 | 386 | –3.1 | 13.9 |  | 504 | –4.2 | 13.7 | 542 | –2.3 | 14.7 |
| Week 40 | 348 | –4.7 | 14.3 | 383 | –2.6 | 13.8 |  | 504 | –3.8 | 14.2 | 528 | –2.0 | 14.2 |
| Week 48 (EOT) | 344 | –4.8 | 15.0 | 376 | –3.5 | 14.7 |  | 503 | –4.0 | 14.9 | 526 | –2.8 | 14.7 |

**Table S6.** Changes in St George’s Respiratory Questionnaire (SGRQ) symptom scores. Values show change from baseline to Week 48 in activity and impact subscores for the per-protocol end-of-treatment (PP EOT) and intent-to-treat (ITT) populations

| SGRQ score | PP EOT | | | ITT | | | |
| --- | --- | --- | --- | --- | --- | --- | --- |
|  | Moxifloxacin  (N = 344)  Mean (SD) | Placebo  (N = 376)  Mean (SD) | p-value^a^ |  | Moxifloxacin  (N = 503)  Mean (SD) | Placebo  (N = 526)  Mean (SD) | p-value^a^ |
| Total | –4.8 (15.0) | –3.5 (14.7) | 0.312 |  | –4.0 (14.9) | –2.8 (14.7) | 0.290 |
| Symptom | –8.2 (23.0) | –3.8 (20.4) | 0.019 |  | –8.2 (23.5) | –3.8 (20.9) | 0.009 |
| Activity | –3.4 (17.5) | –3.5 (19.2) | 0.761 |  | –1.9 (17.6) | –2.4 (18.5) | 0.353 |
| Impact | –4.7 (17.0) | –3.5 (17.5) | 0.459 |  | –4.0 (17.0) | –2.8 (17.5) | 0.331 |

^a^p-values obtained from ANCOVA model, adjusting for baseline score, sex, age, and geographic region.

**Table S7.** Median moxifloxacin minimum inhibitory concentrations (MIC_50_). Values are MICs (numbers) for bacteria isolated from the sputum and rectal swab samples of moxifloxacin- or placebo-treated patients in the per-protocol end-of-treatment (PP EOT) population at each study visit.

|  | | MIC_50_ moxifloxacin-treated patients / MIC_50_ placebo-treated patients  (n moxifloxacin-treated patients / n placebo-treated patients) | | | | | | | | | | |
| --- | --- | --- | --- | --- | --- | --- | --- | --- | --- | --- | --- | --- |
|  |  | Screening | Randomization | Week 8 | Week 16 | Week 24 | Week 32 | Week 40 | Week 48 EOT | 8 weeks after EOT | 16 weeks after EOT | 24 weeks after EOT |
| **Sputum sample** | | |  |  |  |  |  |  |  |  |  |  |
| *Haemophilus influenzae* | | 0.015/0.015  (22/25) | 0.015/0.03  (24/37) | 0.015/0.03  (7/31) | 0.015/0.03  (5/25) | 0.015/0.015  (5/18) | 0.030/0.015  (5/21) | 0.015/0.03  (9/23) | 0.015/0.015  (4/28) | 0.015/0.015  (10/25) | 0.015/0.015  (14/19) | 0.015/0.015  (8/19) |
| *Haemophilus parainfluenzae* | | 0.06/0.06  (27/24) | 0.06/0.06  (27/24) | 0.12/0.06  (22/12) | 0.06/0.06  (19/12) | 0.06/0.06  (12/16) | 0.06/0.06  (15/9) | 0.06/0.06  (10/9) | 0.06/0.03  (10/14) | 0.12/0.06  (9/8) | 0.12/0.03  (8/4) | 0.06/0.06  (12/8) |
| *Moraxella catarrhalis* | | 0.03/0.03  (6/6) | 0.03/0.06  (8/7) | 0.06/0.06  (8/12) | 0.06/0.03  (7/9) | 0.06/0.06  (7/9) | 0.03/0.03  (5/10) | 0.03/0.06  (7/10) | 0.03/0.03  (5/9) | 0.03/0.03  (7/4) | 0.03/0.03  (6/5) | 0.03/0.03  (7/7) |
| *Klebsiella pneumoniae* | | 0.06/0.06  (4/6) | 0.12/0.12  (7/9) | 0.06/0.12  (4/6) | 0.12/0.06  (6/2) | 0.12/0.06  (8/3) | 0.12/na  (4/0) | 0.12/0.5  (8/1) | 0.06/na  (6/0) | 0.12/0.12  (4/2) | 0.12/0.06  (3/2) | 0.12/0.06  (3/3) |
| *Streptococcus pneumoniae* | | 0.12/0.12  (12/8) | 0.12/0.12  (15/17) | 0.25/0.12  (9/12) | 0.12/0.12  (9/6) | 0.12/0.12  (3/9) | 0.12/0.12  (2/10) | 0.12/0.12  (7/11) | 0.12/0.12  (6/12) | 0.12/0.12  (6/9) | 0.12/0.12  (8/8) | 0.12/0.12  (5/5) |
| *Staphylococcus aureus* | | 0.03/0.03  (10/10) | 0.03/0.06  (8/11) | 0.06/0.03  (4/8) | 0.06/0.06  (8/9) | 0.06/0.03  (7/5) | 0.06/0.06  (3/5) | 0.06/0.03  (6/1) | 0.06/0.03  (5/2) | 0.03/0.06  (3/5) | 0.06/0.03  (2/3) | 4.00/0.03  (2/2) |
| *Pseudomonas aeruginosa* | | 0.5/0.5  (9/10) | 1/0.25  (10/8) | 1/2  (11/12) | 1/1  (10/13) | 4/1  (9/9) | 2/1  (7/10) | 2/1  (11/8) | 1/2  (8/12) | 2/2  (10/11) | 1/1  (7/8) | 1/1  (10/12) |
| **Rectal swab^a^** | | |  |  |  |  |  |  |  |  |  |  |
| *Escherichia coli* |  | | 0.5/0.5  (22/26) |  |  | 1/0.5  (24/29) |  |  | 1/0.5  (18/15) |  |  | 4/0.5  (21/18) |
| *Enterococcus faecalis* |  | | 0.25/0.25  (19/15) |  |  | 0.25/0.25  (13/13) |  |  | 0.25/0.25  (13/19) |  |  | 0.25/0.25  (9/14) |
| *Pseudomonas aeruginosa* |  | | 2/2  (5/6) |  |  | 2/2  (2/5) |  |  | 2/0.5  (3/3) |  |  | 1/2  4/5) |
| *Klebsiella pneumoniae* |  | | 0.5/0.12  (7/2) |  |  | 0.12/0.12  (4/2) |  |  | 2/0.5  (1/1) |  |  | 0.25/8  (2/1) |
| *Enterococcus faecium* |  | | 4/2  (7/3) |  |  | 0.25/0.5  (7/5) |  |  | 2/4  (3/3) |  |  | 1/0.25  (1/4) |
| *Staphylococcus* spp. |  | | 1/0.12  (15/14) |  |  | 1/0.25  (8/7) |  |  | 1/0.5  (12/4) |  |  | 2/1  (10/8) |

**^a^**Data for *S. aureus* and *Enterobacter* spp. isolated from rectal swabs are not shown, as there were too few organisms isolated for meaningful interpretation of the data.
